# Fluoroscopy-Assisted Transcervical Removal of a Retained Grill-Brush Bristle: A Case Report

**DOI:** 10.7759/cureus.117990

**Published:** 2026-10-15

**Authors:** Giorgos Sideris, Agapi Skouloudaki, Alexandros Christoulakis, Nikolaos Papadimitriou, Alexander Delides

**Affiliations:** 1 2nd ENT Department, Attikon University Hospital, National and Kapodistrian University of Athens, Athens, GRC

**Keywords:** case report, fluoroscopy, grill-brush bristle, india ink, transcervical approach

## Abstract

Retained grill-brush bristles can be difficult to localize and remove when embedded in cervical soft tissues. The present case report highlights the role of fluoroscopy-assisted transcervical exploration following unsuccessful transoral retrieval. Dye diffusion limited the usefulness of computed tomography (CT)-guided India ink marking, while differences in head position compromised the reliability of CT-based surgical navigation. Removal was achieved through a lateral cervicotomy using C-arm fluoroscopy and clips as radiopaque reference markers, followed by an uneventful recovery. This case illustrates the importance of combining adequate surgical access with reliable intraoperative localization when a deeply embedded cervical bristle cannot be retrieved transorally.

## Introduction

Wire bristles from grill-cleaning brushes may adhere to food and be inadvertently ingested, causing injuries ranging from superficial impaction to perforation and deep tissue infection [1]. As early as 1952, Ballenger et al. described esophageal perforation caused by an ingested wire bristle [2]. Grill-brush bristle injuries accounted for an estimated 1,698 emergency department visits in the United States during 2002-2014 and 3,739 during 2015-2023 [3,4].

Localization and removal can be difficult when bristles become embedded beneath the mucosa or within cervical soft tissues. Computed tomography (CT) helps identify bristles missed on endoscopic examination and define their relationship to surrounding structures, guiding the choice of surgical approach [5,6]. We describe fluoroscopy-assisted transcervical removal of a deeply embedded grill-brush bristle after unsuccessful transoral retrieval.

## Case presentation

A 51-year-old nonsmoking woman with hypertension and well-controlled type 2 diabetes mellitus presented with a five-day history of sore throat and odynophagia and two days of muffled voice. She was afebrile and had no respiratory symptoms. Approximately one month earlier, she had experienced an episode of hemoptysis after eating at a grill restaurant. She was assessed at the emergency department of another hospital at that time, but no abnormalities were identified. Intermittent globus sensation persisted thereafter, although she did not recall swallowing a foreign body.

Flexible fiberoptic laryngoscopy showed marked epiglottic edema without obstruction of the laryngeal inlet. No foreign body was visible. Laboratory investigation tests showed a normal white blood cell count, a mildly elevated C-reactive protein level, and an elevated glucose level (Table 1).

**Table 1:** Laboratory findings. μL: microliter; mg/L: milligrams per liter; mg/dL: milligrams per deciliter.

Test	Result	Reference range
White blood cell count	8.9 × 10³/μL	4.0–11.0 × 10³/μL
C-reactive protein	23.9 mg/L	<5.0 mg/L
Glucose	144 mg/dL	70–99 mg/dL

Neck CT revealed a 1.4-cm right epiglottic abscess and a linear hyperdense foreign body in the right lower posterior pharyngeal wall, behind the posterior laryngeal commissure and cricoid cartilage. A cervical radiograph showed an approximately 3-cm curvilinear metallic object projecting over the fifth cervical vertebra (Figure 1).

**Figure 1:**
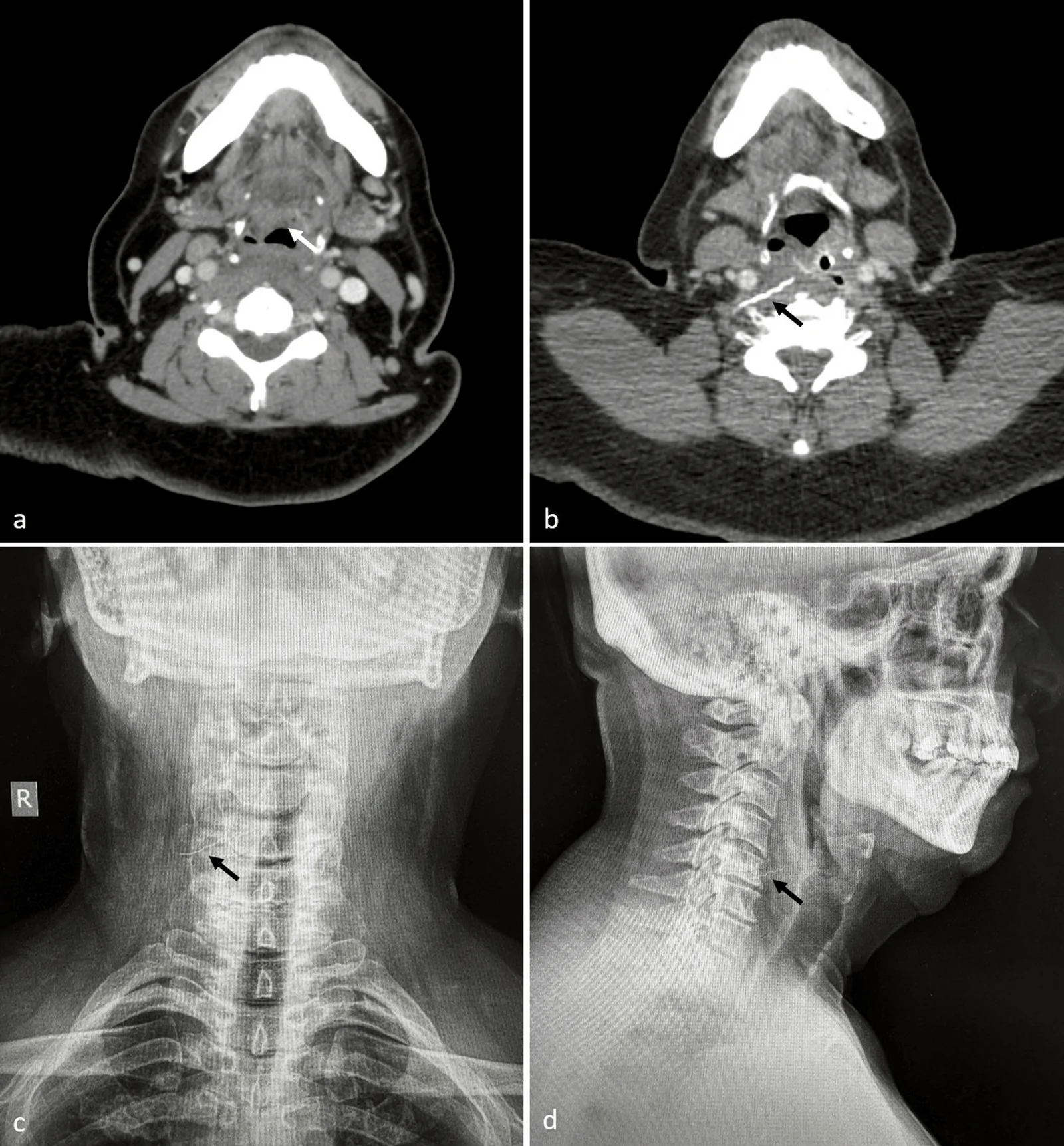
Initial radiological findings. (a) Axial computed tomography showing a small abscess on the lingual surface of the epiglottis (white arrow), located anterior and superior to the retained bristle. The bristle is not visible in this slice. (b) Axial computed tomography showing a linear hyperdense foreign body in the right posterior pharyngeal wall. (c) Anteroposterior and (d) lateral neck radiographs demonstrating the curvilinear metallic bristle at the level of the fifth cervical vertebra. Black arrows in (b–d) indicate the foreign body.

The patient was admitted and received intravenous ceftriaxone (2 g daily), clindamycin (600 mg three times daily), and a single dose of hydrocortisone (250 mg). The epiglottic abscess drained spontaneously. Microlaryngoscopy under general anesthesia was then attempted, but the wire could not be retrieved despite intraoperative C-arm fluoroscopy. A nasogastric tube was placed for enteral feeding. Following the unsuccessful microlaryngoscopic attempt, a second operation through a right lateral cervicotomy was planned. CT-guided India ink marking was performed two hours before the second operation to aid localization (Figure 2).

**Figure 2:**
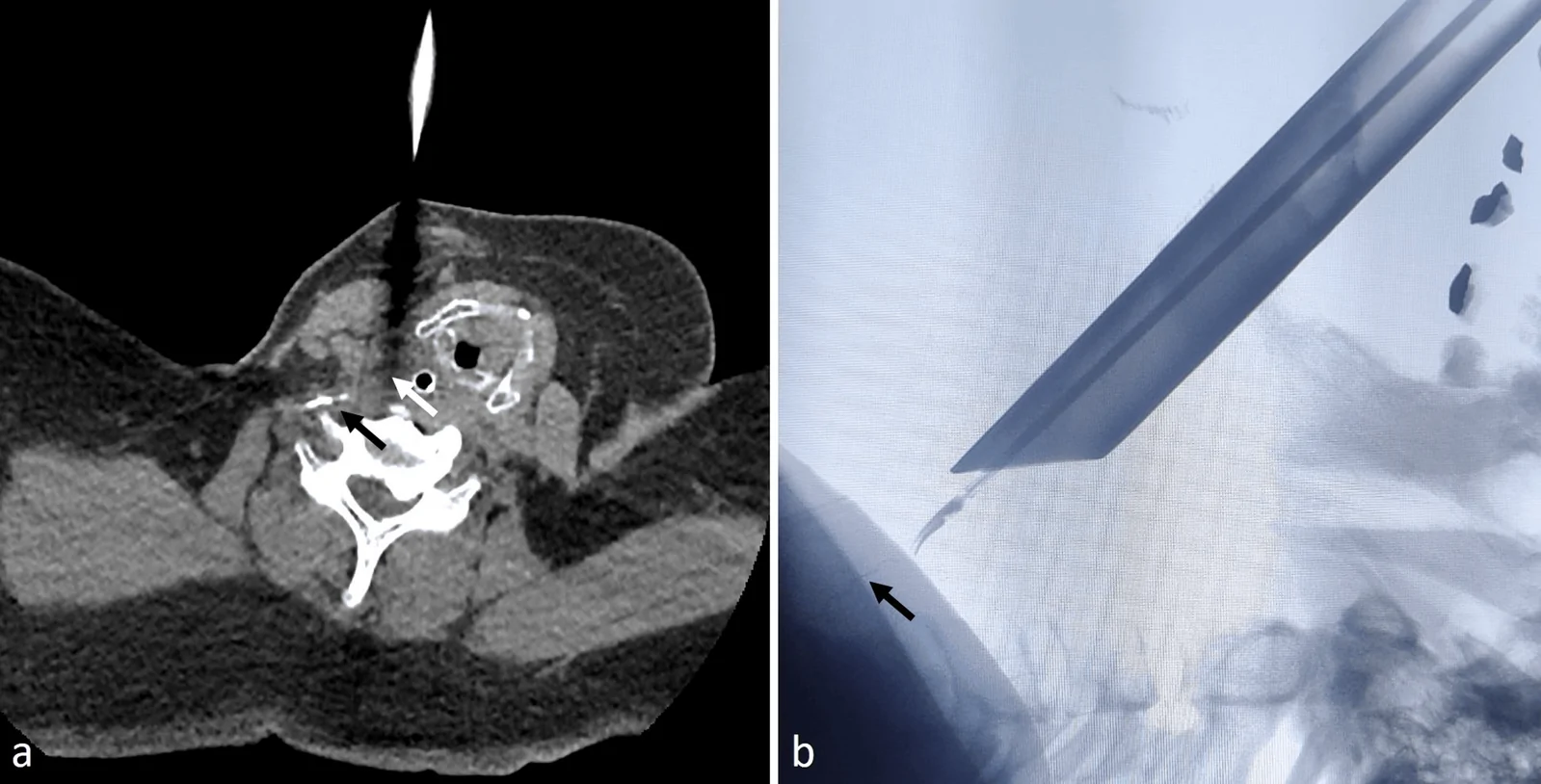
Imaging during localization and attempted retrieval of the retained wire bristle. (a) Axial computed tomography during preoperative India ink marking, showing the marking needle (white arrow) and the retained bristle (black arrow). (b) Intraoperative C-arm fluoroscopy during the unsuccessful microlaryngoscopic retrieval attempt, showing the retained bristle (black arrow).

During exploration through a right lateral cervicotomy, the dye was visible but had diffused into the surrounding tissues and did not accurately indicate the wire’s position. CT-based surgical navigation was attempted but failed to provide reliable localization because the intraoperative head position differed from that during CT acquisition. Clips were used as radiopaque reference markers during intraoperative C-arm fluoroscopy to guide localization of the wire. It was identified in the right lower posterior pharyngeal wall, between the fifth cervical vertebra and the right common carotid artery, and removed. The retrieved foreign body was a bristle from a grill-cleaning brush. A Penrose drain was placed, antibiotics were changed to piperacillin-tazobactam (4.5 g four times daily), and nasogastric feeding was continued (Figure 3).

**Figure 3:**
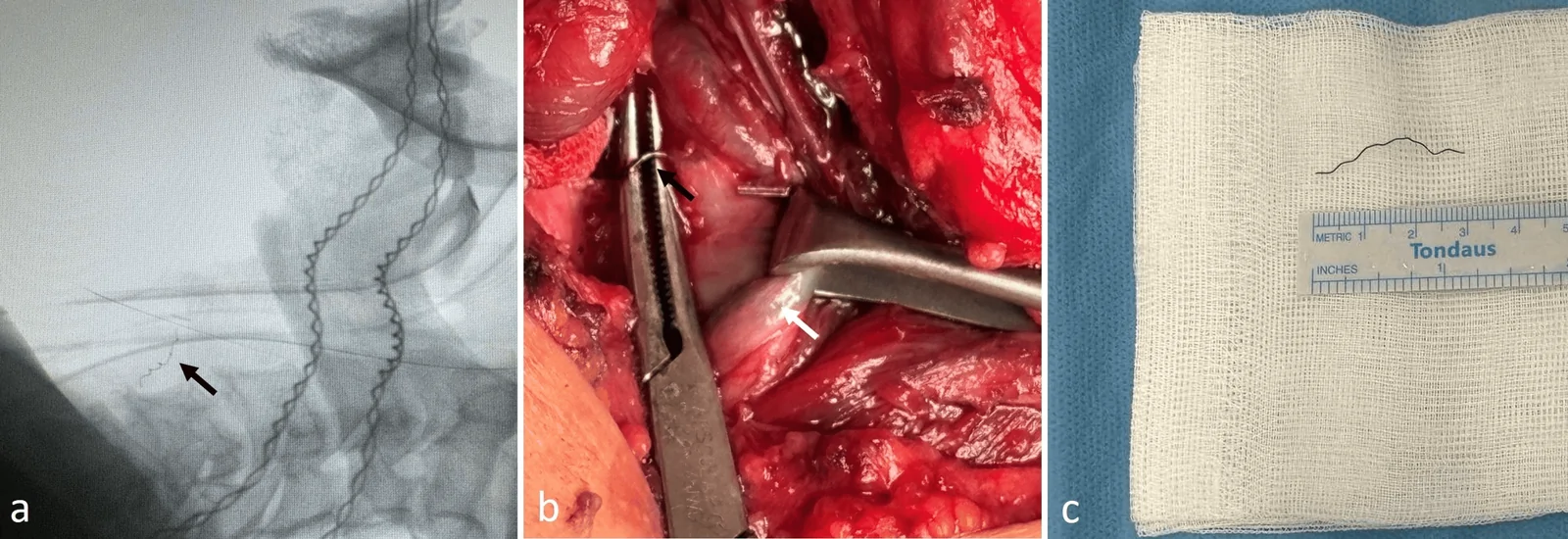
Fluoroscopy-assisted transcervical removal of the retained wire bristle. (a) Intraoperative C-arm fluoroscopy localizing the bristle (black arrow) during transcervical exploration. (b) Operative view through a right lateral cervicotomy showing the bristle held with forceps (black arrow) and the adjacent right common carotid artery (white arrow). (c) Retrieved grill-brush bristle, approximately 3 cm in length.

Recovery was uneventful, with normal vocal fold movement on repeat laryngoscopy. Postoperative flexible endoscopic evaluation of swallowing (FEES) demonstrated satisfactory swallowing function, with no evidence of laryngeal penetration or aspiration. On postoperative day 7, CT with oral contrast showed no evidence of a fistula, and both the nasogastric tube and Penrose drain were removed. The patient was discharged on postoperative day 10.

## Discussion

Two U.S. epidemiological studies illustrate the burden of grill-brush bristle injuries. Using the National Electronic Injury Surveillance System (NEISS), Baugh et al. identified 43 cases during 2002-2014, while Coppola et al. subsequently recorded 85 cases during 2015-2023 [3,4]. These findings highlight the continued clinical relevance of injuries that can pose considerable challenges in diagnosis and removal.

Diagnosis can be difficult when patients do not recall ingestion, and no foreign body is visible on laryngoscopy, as in our case. Wong et al. similarly reported a retained bristle identified on CT after negative endoscopic examinations [6]. In a review of 91 patients by Miller et al., CT identified bristles in 92.8% of reported examinations [5]. Prince et al. also documented migration that changed the planned surgical approach [7]. The small, 1.4-cm abscess was located on the lingual surface of the epiglottis, anterior and superior to the retained bristle in the lower posterior pharyngeal wall at C5. The acute symptoms were consistent with the epiglottic abscess and associated edema. However, the preceding intermittent globus sensation suggested that the bristle may have been causing ongoing local irritation. The retained foreign body may also have acted as a persistent inflammatory stimulus, although its contribution to the epiglottic inflammation and abscess could not be established. Although migration was not demonstrated in our patient, the sharp bristle’s proximity to the right common carotid artery supported removal because of the potential risks of migration, perforation, and injury to adjacent structures [5,7]. The rationale for removal was therefore independent of any presumed relationship with the epiglottic abscess.

The operative approach depends on the bristle’s location and accessibility. Mostafavi and Ganguli used a snare after failed forceps extraction, while Naunheim et al. combined suspension, microscopy, and fluoroscopy for transoral removal of a hypopharyngeal bristle [8,9]. Robotic-assisted removal has also been reported for a tongue-base bristle after unsuccessful conventional laryngoscopy [10]. These reports illustrate the range of techniques available when an initial retrieval attempt fails.

External exploration is an important option when the bristle cannot be reached transorally. Ballenger et al. described cervical mediastinotomy after unsuccessful esophagoscopy [2]. Chen et al. reported neck exploration for a bristle associated with a retropharyngeal abscess [11]. Miller et al. reported successful retrieval in all 12 neck explorations involving bristles initially located in the deep neck [5]. In our patient, unsuccessful microlaryngoscopy prompted a right lateral cervicotomy.

Our case resembles Dalton and Nam’s report of a bristle embedded near the carotid sheath after esophageal perforation. Following unsuccessful endoscopy and initial open exploration, fluoroscopy with needle markers enabled retrieval [12]. In our patient, C-arm fluoroscopy had already been used during the unsuccessful transoral attempt. Its subsequent use during cervicotomy therefore highlights the importance of combining accurate localization with adequate surgical access.

Intraoperative ultrasound has also been used successfully to guide retrieval, as reported by Brooks et al. and Chen et al. [11,13]. In our patient, ultrasound was considered less suitable because of the wire’s deep location. CT-guided India ink marking was performed before the second operation to assist intraoperative localization.

During exploration, however, the dye was visible but had diffused into surrounding tissues and no longer accurately indicated the wire’s position. CT-based surgical navigation was also attempted, but differences in head position between CT acquisition and surgery prevented reliable localization. Clips were then used as radiopaque reference markers during C-arm fluoroscopy, enabling localization and removal of the wire through the external approach.

Although external exploration provides access to deeply embedded foreign bodies, dissection carries risks of bleeding and nerve injury [14]. If the pharyngeal wall is breached, salivary leakage or fistula formation is an additional concern, supported by reports of these complications after lateral pharyngotomy in oncological surgery [15]. These risks reinforce the importance of careful dissection and accurate intraoperative localization. The patient’s diabetes mellitus was another clinical consideration, as this condition has been associated with an increased risk of life-threatening complications in head and neck space infections [16]. In our patient, normal postoperative vocal fold movement, satisfactory swallowing on FEES without penetration or aspiration, and the absence of a fistula on CT with oral contrast documented a favorable early outcome.

This single case cannot establish the comparative safety of different retrieval techniques, and follow-up was limited to hospital discharge.

## Conclusions

Fluoroscopy-assisted transcervical exploration enabled successful removal of a deeply embedded grill-brush bristle after unsuccessful transoral retrieval. CT-guided India ink marking was unreliable because the dye had diffused into surrounding tissues. This case highlights the importance of combining adequate surgical exposure with reliable intraoperative localization in the management of deeply embedded cervical bristles.
